# Psychosocial work characteristic profiles and health outcomes in registered nurses at different stages of their careers: a cross-sectional study

**DOI:** 10.1186/s12913-024-12164-9

**Published:** 2025-02-06

**Authors:** Katri Lönnqvist, Timo Sinervo, Anu-Marja Kaihlanen, Marko Elovainio

**Affiliations:** 1 Doctoral Programme in Population Health, Faculty of Medicine, University of Helsinki, P.O. Box 63, Helsinki, 00014 Finland; 2 Finnish Institute for Health and Welfare, P.O. Box 30, Helsinki, 00271 Finland; 3 Department of Psychology and Logopedics, Faculty of Medicine, University of Helsinki, P.O. Box 21, Helsinki, 00014 Finland

**Keywords:** Organizational justice, Job demand, Job control, Team climate, Health, Registered nurse, Latent profile analysis

## Abstract

**Background:**

Individual psychosocial work characteristics have been associated with the health and well-being of registered nurses. However, it remains to be determined whether different types of psychosocial work characteristics form patterned profiles and whether the profiles are associated with registered nurses’ health and welfare at different stages of their careers*.* The purpose of this study was to identify latent psychosocial work characteristic profiles and examine whether the profiles are associated with a certain career stage and health outcomes.

**Methods:**

This cross-sectional study was conducted with 624 early-career registered nurses and 1,016 later-career registered nurses. Data were collected using an electronic survey with internationally validated measures including the Organizational Justice Scale, the Nurse Stress Index Scale, the Job Content Questionnaire, the Team Climate Inventory, the Psychological Distress Questionnaire, the Sleep Problems Questionnaire, and the Self-Rated Health Questionnaire. Latent profile analysis was conducted to identify subgroups with similar psychosocial work characteristic profiles. Multinomial and linear regression analyses were used to examine the association between latent work characteristics profiles, stage of career, and health outcomes.

**Results:**

We identified five profiles. The profiles were named based on class descriptions. The low strain/high support profile group and the moderate strain/high support profile group had statistically better self-rated health (*p* = < 0.001), less psychological distress (*p* = < 0.001) and less sleep problems (*p* = < 0.001) compared to the high strain/low support profile group.

**Conclusions:**

Low to moderate strain, high interactional and procedural justice, and participative safety in teams form patterned profiles associated with better health in registered nurses. High strain, a lack of justice and a lack of participation safety form a risk combination pattern profile that may lead to health problems in registered nurses. Promoting procedural and interactional justice, and participation safety in teams seems efficient in enhancing the health and well-being of registered nurses. The findings indicate no significant correlation between career stages and work characteristic profiles. It is crucial to identify stressors specific for career stages and develop tailored interventions.

## Background

Psychosocial work characteristics have been widely recognized as playing an important role in employees’ well-being and health [1–6]. Registered nurses (hereinafter referred to as RNs) work in healthcare organizations has been described as particularly stressful in the past few years [7]. Early-career RNs may confront high levels of stress during the transition to working life. Heavy workloads, role conflicts, and a lack of support may contribute to stress. Prolonged exposure to stressors can lead to more severe health problems such as sleep problems during the transition [8]. RNs in mid- and late career stages may experience increased stress due to increasing job demands and the challenge of balancing with work and family life. Moreover, the process of aging can impact both physical and mental health while sleep disturbances and chronic health problems become more prevalent and may impact overall well-being [9, 10]. Stress-related psychosocial factors at work not only have an adverse association with RNs’ health [11], but also associate negatively with the quality of patient care [12] and may lead to turnover intention in early career [13] and early retirement intention in later career stages [14].

Two of the well-established models defining psychosocial work characteristics are the organizational justice model [15] and the job strain model [16, 17]. The organizational justice model refers to the procedures an organization uses in making decisions (procedural justice), the quality of the interpersonal interaction between employees and their organization (interactional justice) and the perceived fairness of reward and resource allocation (distributive justice) [15]. Previous reviews indicate that organizational justice and injustice are associated with employees’ physical and mental health. There is evidence of an association between organizational justice and cardiovascular diseases, minor psychiatric disorders, self-related health, insomnia, sickness absences, and mortality [18–21]. A previous meta-analysis showed an association between the perceptions of unfairness and poorer physical and mental health. This association was strongest with the indicators of strain and employees’ psychological condition [19]. Low organizational justice has more intense negative effects on health in situations involving unpredictability and uncertainty [22] and may affect health outcomes (including sleep problems) up to several years later [23, 24]. Changes in perceived justice have also been found to be associated with health. Reduced interactional justice was associated with an increase in sleep problems, while increased interactional justice was linked to a decline in sleep problems [25]. The association of organizational justice and health has also been studied in RNs [26]. There is evidence of an association between perceived justice and work ability [27] and lower incidence of musculoskeletal symptoms [28, 29], psychological distress [30, 31], moral distress [32], depression [33] and fewer sleep problems [34–36] in RNs. Meanwhile, injustice has been found to be associated with poor self-rated health [37, 38], minor psychiatric morbidity [39], and sleep problems [40] in RNs.

The job strain model includes two main dimensions: job demand (e.g. the need to work quickly and hard) and job control (e.g. control over skill use and time allocation). The job strain model suggested that employees who have concurrent low job control and high demands are unable to moderate stress caused by the high demands. Based on this model, persistent exposure to stress increases the risk of developing health problems [17]. The job strain model has gained empirical support in previous reviews and meta-analyses [1–6]. The job strain hypothesis has been also confirmed in RNs [41]. More recently, elements of the job strain model have been examined in different career stages [42] and have been shown to be associated with health in early career [43, 44] and later career stages in RNs [45].

We also considered other potential effects on the health and well-being of RNs. The team climate model refers to shared perceptions of the organizational environment and factors related to cooperation in a team. One of these factors, participative safety, is characterized by motivated and reinforced involvement in decision-making which occurs in an interpersonally non-threatening environment. According to this model, the more people participate in decision-making, the more likely they are to offer ideas for new and improved ways of working [46]. Team climate has been shown to be associated with psychosocial factors [47] and health outcomes in RNs [48, 49]. A safe team climate has been found to be associated with a lower level of burnout [50, 51], lower level of stress and better well-being [52] in RNs. Meanwhile, a lack of a team climate has been found to be associated with burnout, psychological distress, anxiety, and depression in RNs [52].

A large body of evidence suggests that organizational injustice, job strain, and a lack of team climate at work may lead to health problems [2]. Previous studies on psychosocial work characteristics and RNs’ health have mostly focused on individual psychosocial work characteristics and health outcomes. It has been observed that latent and person-centered analytic approaches may extend the current knowledge and provide more comprehensive knowledge of the psychosocial work environment’s protective or risk factors and their relation to RNs’ health [53]. Examining the patterns of multiple indicators may expand the understanding of how work-related factors influence health and well-being of RNs.The current shortage of RNs requires healthcare organizations to observe working conditions and discover more ways to enable RNs to work healthily throughout their careers. Hence, it would be meaningful to investigate what kinds of latent psychosocial work characteristic profiles can be observed in RNs and whether the profiles are associated with their health and well-being.

The aim of this study was to examine which latent profiles can be observed, to what extent the stages of career are associated with certain profiles, and to what extent these latent profiles improve or deteriorate RNs’ health.

The research questions were:

1. Which work characteristic profiles can be identified in RNs?
2. To what extent is the stage of career associated with work characteristic profiles?
3. To what extent are work characteristic profiles associated with health outcomes?

## Methods

The study included two groups of participants; the first comprised early career RNs (*n* = 6,979) who had up to two years of work experience in the data collection phase. The group comprised all nurses who graduated between the years 2016 and 2018 in Finland. The second group included RNs who had at least two years of work experience (*n* = 10,000). The nurses were randomly selected from the Finnish Central Register of Valvira (the National Supervisory Authority for Welfare and Health). To ensure that the numbers of respondents in each of the two groups matched, the sample size of the more experienced RNs was limited to correspond to that of the early career RNs (while taking possible non-responses into consideration). We obtained the email addresses of 3,942 of the early career RNs and 7,000 of the experienced RNs from the register of the Finnish Association of Health and Social Care Professionals. Nurses whose email addresses could not be obtained were excluded from the study (3,037 early career RNs and 3,000 experienced RNs). The nurses included in the study were sent an invite to take part in the study and received a link to the online questionnaire via email. Information was provided in the invitation letter about the purpose of the study, the voluntary nature of filling out and submitting the questionnaire, and the fact that data will be processed anonymously and only by the research team. Data collection took place between 1 November and 21 December 2018. After this process and three subsequent email reminders, a total of 712 early career RNs and 1,226 experienced RNs responded to the survey. After removing missing data, the results of 624 (15.83%) early career RNs and 1,016 (14.51%) experienced RNs were analyzed.

### Measures

Seven previously validated self-report scales including the Organizational Justice Scale, the Nurse Stress Index Scale, the Job Content Questionnaire, the Team Climate Inventory, the Psychological Distress Questionnaire, the Sleep Problems Questionnaire, and the Self-Rated Health Questionnaire were adopted for assessment purposes in this study.

*Organizational justice* was measured using an 8-item scale (e.g.,*”The procedures used in my organization have been applied consistently”*) derived from the short version of the Organizational Justice Scale [54, 55] measuring procedural (3 items), interactional (3 items), and distributive justice (2 items). The items were rated on a five-point Likert scale ranging from 1 (fully disagree) to 5 (fully agree). The Cronbach’s alphas were 0.80 (procedural justice), 0.93 (interactional justice), and 0.91 (distributive justice) in this study.

*Job demand (workload)* was measured using a 3-item scale (e.g.,*”How often has constant rush and pressure due to uncompleted work disturbed, worried or stressed you during the last two months”)* derived from the Nurse Stress Index [56] measuring excessive workloads and time pressures. The items were rated on a five-point Likert scale ranging from 1 (hardly ever) to 5 (very often or continuously). The Cronbach’s alpha was 0.90 in this study.

*Job control* was measured using a 3-item scale (e.g.,*”My job allows me to make a lot of decisions of my own”)* derived from the Job Content Questionnaire [57] measuring the freedom to make independent decisions. The items were rated on a five-point Likert scale ranging from 1 (fully disagree) to 5 (fully agree). One item was reverse-coded. The Cronbach’s alpha was 0.69 in this study.

*Team climate* was measured using a 4-item scale (e.g., *“People keep each other informed”*) derived from the short version of the Team Climate Inventory measuring participation safety [46, 58]. The items were rated on a five-point Likert scale ranging from 1 (fully disagree) to 5 (fully agree). The Cronbach’s alpha was 0.88 in this study.

*Psychological distress* was measured using a 4-item scale *(e.g., “Have you recently felt constantly under strain?”)* derived from the General Health Questionnaire [59]. The items were rated on a four-point Likert scale ranging from 1 (not at all) to 4 (a lot more than usually). The Cronbach’s alpha was 0.86 in this study.

*Sleep problems* were measured using a 4-item scale (e.g.,”*How often during the past weeks have you e.g. woken up feeling tired and worn out after the usual amount of sleep”*) derived from the Sleep Problems Questionnaire [60]. The items were rated on a six-point Likert scale ranging from 1 (not at all) to 6 (every night). The Cronbach’s alpha was 0.83 in this study.

Self-rated health was measured using a 1-item scale (*How is your health compared to other people your age?* [61]. The item was rated on a five-point Likert scale ranging from 1(good) to 5 (poor).

### Data analysis

We used the Latent Profile Analysis (LPA), a person-centered approach that aims to identify subgroups of individuals who share similar profiles of scores on various indicators of interest [62]. Following the six procedures suggested by Ferguson et al. [63], we started by first assessing the profile indicator variables and measured the mean value variables of procedural justice, interactional justice, distributive justice, job demands, job control, and team climate. Second, we used the Akaike information criterion (AIC), Bayesian Information Criterion (BIC), the Sample-Adjusted BIC (SABIC) and an entropy test for our model selection. Third, we used a step-by-step approach to define the number of latent profiles most descriptive of the data set and sample, by first conducting an LPA with one profile and subsequently adding the profiles. Fourth, we investigated our findings based on a suggestion according to which the lower values of AIC, BIC, and SABIC indicate a better fit for the model. The entropy test values ranged from 0 to 1, with higher values indicating a better differentiation between profiles [64, 65]. Fifth, multinomial logistic regression was used in the analysis to examine the associations of the stage of career and other individual and structural characteristics with profile class membership. Linear regression analysis was used to examine the association between profile class memberships and psychological distress, sleep problems, and general health and in an age- and sex-controlled manner. Lastly, analyses were conducted using the R statistical software, version 4.2.2 utilizing the Lavaan, TidyLPA, Tidyverse, Dplyr, Epikit, Mclust, Ggplot, Nnet, Sjplot, and Sjmisic packages [66].

## Results

Based on the lowest values of the AIC, the six-class model offered the best fit and lowest values of the BIC and the SABIC, the five-class model offered the best fit. The entropy test value offered the best fit for the three-class model (Table 1).

**Table 1:** Model selection

Classes	AIC	BIC	SABIC	Entropy
1	27412.491	27477.321	27439.199	1.000
2	25773.279	25875.926	25815.566	0.778
3	25243.850	25384.313	25301.716	0.804
4	24950.966	25129.247	25024.412	0.762
5	24804.075	25020.173	24893.099	0.770
6	24792.756	25046.671	24897.360	0.708

*AIC* Akaike Information Criterion, *BIC* Bayesian Information Criterion, *SABIC* Sample size-adjusted Bayesian Information Criterion, *Entropy* Entropy test

**Fig. 1:**
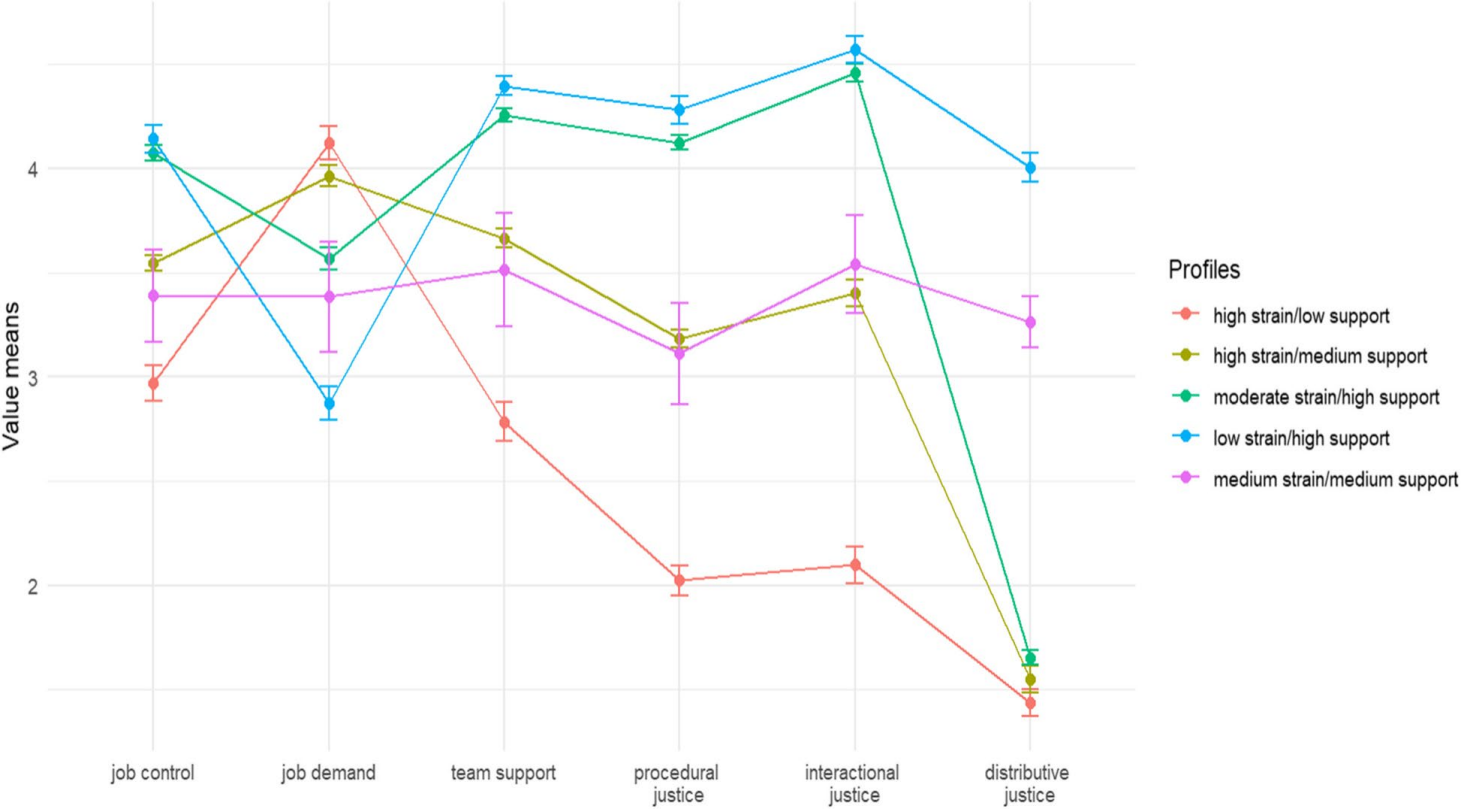
The class descriptions of psychosocial work characteristics profiles

We selected the five-class model as the optimal choice based on low BIC and SABIC values and the relatively small difference in the AIC values between the five-class and six-class models and in the entropy test value between three-class and five-class models [62, 63]. The class descriptions are based on comparisons between psychosocial work characteristic indicators including job control, job demand, team climate, procedural justice, interactional justice, and distributive justice. These indicators are presented as mean values and standard deviations (Fig. 1). The profiles were named based on the descriptions of classes as high strain/low support, high strain/medium support, moderate strain/high support, low strain/high support, and medium strain/medium support.

The high strain/low support profile had a low level of job control, a high level of job demand, a low level of team support, and a low level of organizational justice in all three indicators. The high strain/medium support profile was characterized by a medium level of job control, a high level of job demand, a medium level of team support, a medium level of procedural and interactional justice, and a low level of distributive justice. The moderate strain/high support profile was characterized by high job control, moderate job demand, high procedural and interactional justice, and low distributive justice. The low strain/high support profile was characterized by high job control, low job demand, high team support, and a high level of organizational justice in all three indicators. The medium strain/medium support presented a medium level of job control and job demand, a medium level of team support, and a medium level of organizational justice in all three indicators.

The personal and work-related characteristics of the RNs are summarized in Table 2. Most participants in the total sample were female (91%) and their mean age was 39. Most of the participants had been working in three shifts, in a hospital, and in the public sector. Because it is highly probable that career stage and age are correlated, we repeated the regression analysis in two age groups (under 40 years and 40 years or over). The results showed that distress was statistically significantly (*p* = < 0.001) associated with age group under 40 years and general health was statistically (*p* = < 0.020) associated with age group under 40 years. Statistical difference in sleep problems between these two age groups was not observed (*p* = 0.937). Profile groups were formed as follows: The high strain/low support (*n* = 206, 12.56%), the high strain/medium support (*n* = 546, 33.29%), the moderate strain/high support (*n* = 501, 30.55%), the low strain/high support (*n* = 241, 14.70%), and the medium strain/medium support (*n* = 146, 8.90%). Most of the participants in the high strain/low support profile, the high strain/medium support, and the moderate strain/high support groups had been working as nurses for over two years. The low strain/high support profile and the medium strain/medium supporr profile groups had nearly identical numbers of both early career RNs and experienced RNs. Belonging to the low strain/high support profile was statistically significant in early career RNs who had worked less than two years compared to belonging to the high strain/low support profile (Table 3).

**Table 2:** RNs’ personal and work characteristic variables

	Entire sample	High strain/low support	High strain/medium support	Moderate strain/high support	Low strain/high support	Medium strain/medium support
n (%)	1640	206 (12.56)	546 (33.29)	501 (30.55)	241 (14.70)	146 (8.90)
Stage of career						
≤ 2 years	624	51 (24.76)	205 (37.55)	188 (37.52)	118 (48.96)	62 (42.47)
˃2 years	1016	155 (75.24)	341 (62.45)	313 (62.48)	123 (51.04)	84 (57.53)
Sex						
Male	134 (8.17)	12 (5.83)	36 (6.59)	38 (7.58)	34 (14.11)	14 (9.59)
Female	1506 (91.83)	194 (94.17)	110 (93.41)	463 (92.42)	207 (85.89)	132 (90.41)
Age						
20–29	514 (31.34)	37 (17.96)	186 (34.07)	161 (32.14)	81 (33.61)	49 (33.56)
30–39	357 (21.77)	56 (27.18)	119 (21.79)	90 (17.96)	55 (22.82)	37 (25.34)
40–49	349 (21.28)	57 (27.67)	111 (20.33)	115 (22.95)	46 (19.09)	20 (13.70)
50–	420 (25.61)	56 (27.18)	130 (23.81)	135 (26.95)	59 (24.48)	40 (27.40)
Work schedule						
One-shift	645 (39.33)	87 (42.23)	197 (36.08)	218 (43.51)	94 (39)	49 (33.56)
Two-shift	269 (16.40)	35 (16.99)	76 (13.92)	78 (15.57)	45 (18.67)	35 (23.97)
Three-shift	650 (39.63)	74 (35.92)	249 (45.60)	187 (37.33)	84 (34.85)	56 (38.36)
Other	76 (4.63)	10 (4.85)	24 (4.40)	18 (3.59)	18 (7.47)	6 (4.11)
Work experience in current unit						
1–11 months	522 (31.83)	54 (26.21)	166 (30.40)	156 (31.14)	97 (40.25)	49 (33.56)
1–5 years	531 (32.38)	61 (29.61)	181 (33.15)	160 (31.94)	77 (31.95)	52 (35.62)
6–15 years	298 (18.17)	55 (26.70)	100 (18.32)	92 (18.36)	29 (12.03)	22 (15.07)
over 15 years	289 (17.62)	36 (17.48)	99 (18.13)	93 (18.56)	38 (15.77)	23 (15.75)
Unit						
Elderly care	107 (6.52)	16 (7.77)	31 (5.68)	32 (6.39)	21 (8.71)	7 (4.79)
Hospital	761 (46.40)	95 (46.12)	269 (49.27)	237 (47.31)	97 (40.25)	63 (43.15)
Outpatient	674 (41.10)	83 (40.29)	225 (41.21)	207 (41.32)	100 (41.49)	59 (40.41)
Other	98 (5.98)	12 (5.83)	21 (3.85)	25 (4.99)	23 (9.54)	17 (11.64)
Sector						
Public	1366 (83.29)	174 (84.47)	475 (87)	431 (86.03)	171 (70.95)	115 (78.77)
Private	228 (13.90)	27 (13.11)	59 (10.81)	61 (12.18)	54 (22.41)	27 (18.49)
Third	46 (2.80)	5 (2.43)	12 (2.20)	9 (1.80)	16 (6.64)	4 (2.74)

**Table 3:** The association between stage of career, personal and work characteristics to class profiles

	High strain/medium support vs. high strain/ low support			Moderate strain/high support vs. high strain/ low support			Low strain/high support vs. high strain/low support			Medium strain/medium support vs. high strain/low support		
Predictors	OR	CI	*p*	OR	CI	*p*	OR	CI	*p*	OR	CI	*p*
(Intercept)	5.81	1.04–32.56	0.045	8.75	1.58–48.62	0.013	27.33	4.39–170.08	< 0.001	2.65	0.31–22.97	0.376
Career (≤ 2 yrs = ref.)	0.66	0.42–1.03	0.069	0.55	0.35–0.87	0.011	0.34	0.21–0.58	< 0.001	0.48	0.27–0.85	0.012
Sex (Men = ref.)	0.92	0.46–1.83	0.811	0.74	0.38–1.48	0.400	0.36	0.17–0.73	0.005	0.59	0.26–1.35	0.214
Age	0.98	0.96–1.00	0.023	0.99	0.97–1.01	0.326	1.00	0.98–1.02	0.830	0.99	0.97–1.01	0.428
Work experience in current unit												
1–11 months	ref.	ref.	ref.	ref.	ref.	ref.	ref.	ref.	ref.	ref.	ref.	ref.
1–5 years	1.13	0.73–1.75	0.594	1.05	0.68–1.65	0.814	0.85	0.52–1.40	0.534	1.10	0.63–1.93	0.731
6–15 years	1.03	0.60–1.76	0.919	0.87	0.51–1.50	0.622	0.47	0.25–0.92	0.027	0.73	0.35–1.53	0.407
over 15 years	1.84	0.98–3.45	0.059	1.45	0.77–2.73	0.248	1.22	0.59–2.53	0.597	1.45	0.63–3.35	0.387
Work schedule												
One-shift	ref.	ref.	ref.	ref.	ref.	ref.	ref.	ref.	ref.	ref.	ref.	ref.
Two-shift	1.01	0.61–1.68	0.976	0.82	0.49–1.36	0.446	1.10	0.62–1.96	0.741	1.99	1.06–3.73	0.033
Three-shift	1.43	0.86–2.39	0.164	0.77	0.46–1.28	0.309	1.07	0.59–1.93	0.817	1.42	0.74–2.79	0.290
Other	1.09	0.46–2.59	0.847	0.57	0.23–1.40	0.218	1.45	0.56–3.74	0.439	1.04	0.32–3.36	0.952
Current work unit												
Elderly care	ref.	ref.	ref.	ref.	ref.	ref.	ref.	ref.	ref.	ref.	ref.	ref.
Hospital	0.99	0.48–2.04	0.972	1.19	0.58–2.46	0.631	0.83	0.37–1.98	0.662	1.50	0.53–4.23	0.441
Outpatient	1.38	0.69–2.74	0.360	1.16	0.59–2.30	0.665	1.21	0.57–2.60	0.619	2.20	0.82–5.92	0.117
Other	0.99	0.38–2.60	0.987	1.14	0.44–2.92	0.787	1.81	0.65–5.00	0.253	5.34	1.60–17.87	0.007
Sector												
Private	ref.	ref.	ref.	ref.	ref.	ref.	ref.	ref.	ref.	ref.	ref.	ref.
Public	1.12	0.67–1.88	0.656	1.04	0.62–1.73	0.889	0.48	0.28–0.83	0.009	0.64	0.35–1.20	0.165
Third	1.24	0.39–3.97	0.720	0.86	0.26–2.88	0.806	1.45	0.46–4.61	0.528	0.59	0.13–2.57	0.480

The low strain/high support profile had statistically significantly better self-rated health (*p* = < 0.001), less sleep problems (*p* = < 0.001), and less psychological distress (*p* = < 0.001) compared to the high strain/low support profile group. The moderate strain/high support profile had statistically significantly better self-rated health (*p* = < 0.001), less sleep problems (*p* = < 0.001), and less psychological distress (*p* = < 0.001) compared to the high strain/low support profile group. Self-rated health, sleep problems, and psychological distress were statistically better in those in the high strain/medium support group and the medium strain/medium support group when compared to the high strain/low support profile (Table 4).

**Table 4:** The associations of class profiles to health outcomes

	Distress			Sleep problems			General health		
Predictors	Est.	CI	*p*	Est.	CI	*p*	Est.	CI	*p*
Interept	2.48	2.29–2.68	< 0.001	2.85	2.54–3.15	< 0.001	1.74	1.50–1.98	< 0.001
Age	−0.01	−0.01– 0.00	< 0.001	0.00	−0.00–0.01	0.678	0.01	0.00–0.01	0.002
Sex	0.15	0.02–0.28	0.020	0.19	−0.02–0.39	0.071	0.14	−0.02–0.30	0.092
High strain/low support	ref.	ref.	ref.	ref.	ref.	ref.	ref.	ref.	ref.
High strain/medium support	−0.21	−0.32– −0.09	0.001	−0.20	−0.38– −0.02	0.030	−0.17	−0.31– −0.03	0.010
Moderate strain/high support	−0.44	−0.56– −0.32	< 0.001	−0.45	−0.64– −0.27	< 0.001	−0.27	−0.42– −0.13	< 0.001
Low strain/high support	−0.68	−0.82– −0.54	< 0.001	−0.81	−1.02– −0.60	< 0.001	−0.46	−0.62– −0.29	< 0.001
Medium strain/medium support	−0.23	−0.39– −0.08	0.003	−0.33	−0.57– −0.09	0.007	−0.26	−0.45– −0.07	0.007

*Est*. Estimate, *CI* Confidence intervals

## Discussion

The purpose of this study was to identify latent work characteristic profiles including job demand, job control, team support, procedural justice, interactional justice, and distributive justice, and to examine whether the profiles were associated with different stages of career as well as health outcomes. We used LPA to expand the previous knowledge of psychosocial work environments’ protective factors and high-risk factors to RNs’ health. Five distinct profiles guided by the organizational justice model [15], job strain model [16, 17], and team climate model [46] emerged from our analyses. Previous LPA studies recommended that both statistical fit values as well as theoretically coherent and content-related aspects should be considered when selecting profile groups [64]. Based on these deliberations, LPA provided novel insight into how the work characteristic profiles are patterned and which profile patterns improve or deteriorate RNs’ health.

According to our results, distress, sleep problems, and poor overall health were more common in the high strain/low support group compared to the moderate strain/high support group and the low strain/high support group. In previous studies, job control has been found to serve as a protective factor mitigating the negative impact of work demands [16]. For example, Parizad et al. [67] pointed out in their study that job control decreases nurses’ job stress and improves collaboration with other professionals. Moreover, it has been suggested that psychological demands and work efforts were associated with higher levels of burnout and lower levels of self-rated health whereas social support was associated with lower levels of burnout and better self-rated health in RNs [68]. When faced with uncertainty (e.g. due to no job control), employees pay close attention to how fair the decisions made by their manager are. Employees find fairness important as it facilitates their coping with uncertainty [22]. The members in the high strain/low support group were mostly later career RNs with over two years of work experience. Work experience usually brings more duties and responsibilities and may lead to intense stressful circumstances for experienced RNs. Factors related to a negative work environment, such as demanding duties or multi-tasking, can trigger negative effects on employees. High demands may lead to stress in the long term. Stressed employees struggle more with using adaptive coping strategies, such as prioritizing their tasks and reorganising their duties. Stressed employees may feel or actually be less able to cope at work [69]. In stressful times, fairness plays a key part for employees as it promotes their ability to cope with uncertainty [22]. Moreover, the association between age and work-related stress has been studied as it has been believed that age brings balance to stress management. However, mixed results have been obtained regarding the effects of age on perceived work-related stress [70]. Perceived sleep quality, distress and self-rated health were substantially worse in the high strain/low support profile group. This finding is consistent with previous studies as strain and injustice have been found to be associated with negative health outcomes such as sleep problems [34–36] and distress [30, 31] in RNs. Previous research has also shown that the negative health effects of low organizational justice are more intense in situations involving unpredictability and uncertainty [22]. Moreover, a lack of team climate has been found to be associated with poorer psychological health in RNs [52].

The members in the low strain/high support group and the moderate strain/high support group presented with better self-rated health, sleep quality, and less psychological distress. This finding is in line with previous studies in which high organizational justice [21], participation safety in teams [51], and low and moderate job strain (1) were also associated with better health in RNs. Early career somewhat predicted membership in the low strain/high support group compared to the high strain/low support profile group. Previous studies have shown that heavy workloads and a lack of competence are sources of stress in the early career stage of RNs [71, 72]. However, a supportive team climate decreases stress [50] and interactional and procedural justice have been shown to be associated with better health outcomes in early career RNs [36]. The members in the high strain/medium support group exhibited better self-rated health and reported fewer sleep problems and less psychological distress than those in the high strain/low support profile. This suggests that even moderate levels of support can serve as a buffer against the detrimental health effects of high work strain, reinforcing previous research that emphasizes the protective role of support in high-stress work environments [73]. Furthermore, the medium strain/medium support group showed similar advantages, further supporting the idea that a balance between manageable strain and adequate support may help mitigate negative health outcomes. This is consistent with the previous studies [73].

The RNs’ experiences of low distributive justice were highly similar in the high strain/low support profile, the high strain/medium support profile, and the moderate strain/high support profile. This finding may partly be explained by the perception that employees who experience injustice because of stress at work also consider their pay and rewards unfair. By contrast, positive work environmental factors may mitigate the effects of negative work-related factors, such as low pay, on the overall perception of the psychosocial work environment [74]. On the other hand, our finding can also be partly explained by the different expectations for working life that RNs have in various career stages. Overall, nurses’ salaries can be considered low compared to the demands faced by the nursing workforce in their work [75], but individuals may have different perceptions of the importance of pay [74]. Other psychosocial work quality factors, such as autonomy, being part of a well-functioning team, fair and supportive leadership, work-life balance, and career opportunities may be more significant factors for RNs at different stages of their career [47]. Meanwhile, a lack of psychosocial work quality factors may increase stress and lead to intent to leave or actual turnover [44] and thus, retention strategies should be adapted to the changing needs of RNs at different stages of their careers [76].

Nurse managers should consistently evaluate the well-being of RNs through surveys, feedback sessions, team meetings, or informal check-ins. This may help in identifying early signs of stress and provide opportunities for timely and more tailored interventions and support systems. Early-career RNs may benefit from tailored orientation programs and structured support to facilitate their transition into the workforce. In contrast, mid-career RNs may require resources that assist in balancing work and family responsibilities, while also promoting autonomy in their roles. In-late career RNs, interventions could be directed toward strategies to maintain physical and mental well-being, such as implementing flexible work schedules. By focusing on practical strategies, nurse managers can promote a supportive and fair environment that not only reduces uncertainty and stress but also enhances RNs’ overall well-being at different stages of career. Comprehending the relationship between nurses' career stages and their health is essential for the development of targeted interventions that promote RNs well-being throughout their professional career [77].

### Limitations

This cross-sectional study does not lend itself to making interpretations of the direction of the associations or causal relationships between psychosocial work characteristics and health outcomes. The response rate of 15.83% (early career RNs) and 14.51% (experienced RNs) appears somewhat low and as a result, this sample may not be representative of the population as a whole. However, these response rates can be considered acceptable [78] and the sample sizes were sufficient. The present study includes a limited range of psychosocial work characteristics and other factors that may be associated with RNs’ health and well-being. However, this study provides an overview of the profiles of RNs working in different career stages, work shifts, work units, and healthcare sectors. It is also worth pointing out that the research data were collected before the COVID-19 pandemic. We cannot rule out the possibility that the results might have been different if the data had been obtained after the pandemic. However, it can be assumed that all the policies and practices supporting RNs’ physical and mental health are still key elements in healthcare organizations and everyday management [47].

## Conclusions

The findings indicate that a profile characterized by low strain and high support may serve as a protective factor against health issues such as distress and sleep problems, while promoting overall health. Conversely, a profile characterized by high strain and low support represents a combination of risk factors that may contribute to health problems. Based on the findings, there appears to be no significant association between career stages and work characteristic profiles. Instead, it is essential to identify the stressors specific to different career stages and develop tailored solutions.

Further research is needed on whether job demand-control and organizational justice factors, team climate, and other individual-, work- and health-related outcomes are patterned and whether the improvement of job demand-control, organizational justice, and team climate results in better long-term health. Moreover, further research could build upon findings in this study by applying Latent Profile Analysis (LPA) to larger and more diverse nurse populations to validate the identified profiles and examine their temporal stability. It would also be valuable to explore whether certain profiles demonstrate differential responsiveness to specific organizational interventions or support systems.

## Data Availability

The datasets used and/or analysed during the current study are available from the authors on reasonable requests.

## Abbreviations

AIC: Akaike Information Criterion
BIC: Bayesian Information Criterion
CI: Confidence Interval
LPA: Latent Profile Analysis
RN: Registered nurse
SABIC: Sample-Adjusted BIC
